# Groundwater Driving Factors Identification and Its Associated Human Health Risk Assessment in a Metropolitan City of Southwest China

**DOI:** 10.3390/toxics14010019

**Published:** 2025-12-24

**Authors:** Xiaoyan Zhao, Huan Luo, Rongwen Yao, Zhan Xie, Si Chen, Lizhou Zhang, Yunhui Zhang, Yangshuang Wang, Yang Liu

**Affiliations:** 1 Faculty of Geosciences and Engineering, Southwest Jiaotong University, Chengdu 611756, China; 2 Yibin Research Institute, Southwest Jiaotong University, Yibin 644000, China; 3 Sichuan Province Engineering Technology Research Center of Ecological Mitigation of Geohazards in Tibet Plateau Transportation Corridors, Chengdu 611756, China; 4 Observation and Research Station of Ecological Restoration for Chongqing Typical Mining Areas, Ministry of Natural Resources, Chongqing Institute of Geology and Mineral Resources, Chongqing 401120, China; 5 Chongqing Academy of Surveying and Mapping, Chongqing 401120, China

**Keywords:** groundwater, human health risk, hydrochemistry, kernel density method

## Abstract

Health risks associated with groundwater deterioration have become increasingly prominent worldwide. Accurate assessment of human health risks associated with groundwater is a critical component of groundwater development and utilization, particularly in large metropolitan areas with high water resource demands. In our study, 37 groundwater samples were collected from the main urban areas of Chongqing, the largest city in southwest China, to identify the groundwater driving factors and their associated human health risk. The primary hydrochemical facies in the study area is Ca–HCO_3_. Groundwater hydrochemistry is primarily controlled by silicate weathering, carbonate (dolomite and calcite) dissolution, and anthropogenic activities such as industrial and agricultural activities. The hazard index (HI) caused by NO_3_^−^ and NO_2_^−^ was higher than the safety standard and exhibited potentially noncarcinogenic risk for children in the north and the west of the study area. The KDE-based Monte Carlo simulation method showed a high reliability in human health risk assessment, with all mean values of the original dataset falling within their corresponding 95% confidence intervals (CIs) of generated data. The achievement can provide valuable insights for groundwater risk mitigation and resource management in Chongqing’s main urban areas, as well as in other metropolitan regions worldwide.

## 1. Introduction

Groundwater, as a critical component of global water resources, serves as the primary source of drinking water for approximately 50% of the world’s population and supports nearly 40% of agricultural irrigation demands [1,2,3]. Over the past years, the rapid advancement of urbanization, industrialization, and agricultural expansion has significantly intensified groundwater exploitation, especially in urban areas [4,5,6]. Owing to its inherent vulnerability, groundwater is highly susceptible to contamination during development and utilization [7,8,9]. Therefore, the driving factors and their potential health risks have become key issues that urgently need to be addressed for the sustainable development of the metropolitan cities and the health security of residents [10,11,12].

Determining the chemical characteristics of groundwater is a critical component in conducting health risk assessments and managing groundwater resources [13,14]. Groundwater chemistry is primarily influenced by a combination of natural processes and anthropogenic activities [6,15,16]. Natural processes in groundwater can generally be identified through geological modeling (such as saturation index), specific ionic ratio diagrams (e.g., Gibbs and Gaillardet diagrams), and integration with regional geological conditions [5,17]. Some conservative ions (such as chloride), which are not affected by chemical, physical, or biological processes in the hydrological system, are commonly utilized for anthropogenic activities identification [18,19]. Moreover, the interpolation methods and the land use maps could assist in identifying natural processes and human activities [20,21].

Human health risks caused by contaminants in groundwater are usually evaluated by the health risk model proposed by the United States Environmental Protection Agency (USEPA) [22,23,24]. This model specifically assesses both noncarcinogenic and carcinogenic risks for various population groups exposed to groundwater contaminants [25,26,27]. However, the use of deterministic parameter values within this model may introduce uncertainties into the risk assessment process, primarily arising from the inherent variability and stochastic nature of key input parameters under real-world environmental conditions [12,28]. Monte Carlo simulation techniques, which generate numerous datasets based on assigned probability distributions, can help reduce these uncertainties in health risk evaluations [29,30]. Nevertheless, outliers in the original data may lead to overfitting or underfitting of the probability distributions in Monte Carlo simulations, thereby reducing the reliability of health risk assessments [31,32,33]. Therefore, in this study, a nonparametric Monte Carlo simulation based on the kernel density estimation method was applied to accurately capture the characteristics of pollutant concentration distributions and achieve a more reliable human health risk assessment.

Chongqing is the largest metropolitan area in southwest China, with a population of over 31.9 million people [15,34]. Groundwater serves as the primary source of water supply in the main urban areas of Chongqing and may pose risks to human health due to intensive anthropogenic activities [35,36]. The hydrochemistry and groundwater quality in the main urban area have been comprehensively investigated in previous studies [5,34,35,37]. Other studies have also focused on the spatial distribution of typical contaminants (e.g., nitrate, Fe, and Mn) in Chongqing [21,38]. However, human health risks across the main urban area remain poorly understood. As a result, health risk assessment of groundwater has become an urgent priority for groundwater management in Chongqing. Thus, a comprehensive human health risk assessment was conducted in our study, combined with a traditional deterministic model and a novel probabilistic model based on the kernel density method, to signify the human health risks posed by normal hazards in groundwater [23,39,40,41]. This study aims to (1) identify the driving factors controlling groundwater chemistry in the main urban area of Chongqing, (2) evaluate human health risks using both deterministic and probabilistic methods, and (3) validate the reliability of the probabilistic method for human health risk assessment. Our findings can provide valuable insights for groundwater risk mitigation and resource management in Chongqing’s main urban areas, as well as in other metropolitan regions worldwide.

## 2. Study Area

**Figure 1:**
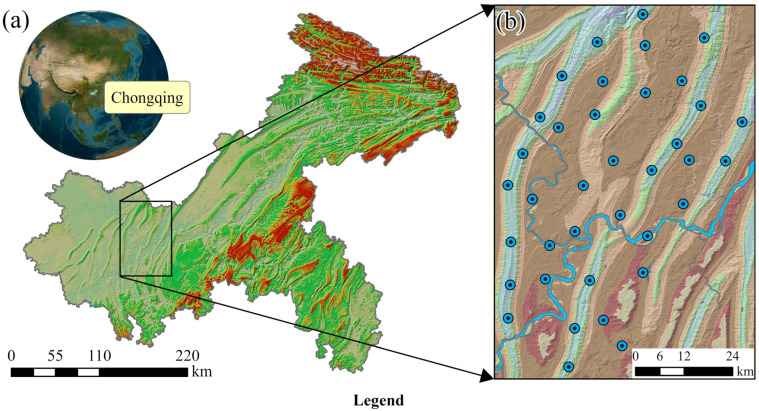
The location of the study area: (**a**) Chongqing in the world and (**b**) the study area in Chongqing [5].

The study area is located in the western part of Chongqing Municipality, Southwest China, encompassing the main urban district with a total area of approximately 4780 km^2^ and a population of about 10.34 million (Figure 1a). The region experiences a subtropical humid monsoon climate, characterized by distinct seasonal variations with an annual mean temperature of 19.4 °C and an average annual precipitation of approximately 1030 mm [35]. Topographically, the terrain descends from northwest to southeast and is dominated by a series of parallel ridges and intermontane valleys trending NE–NNE. Two major rivers, the Jialing River and the Yangtze River, flow through the area from north to south and from south to north, respectively (Figure 1b). The land use pattern is primarily dominated by urban and industrial land concentrated in the valley areas, whereas forest, shrub, and grassland are mainly distributed along the ridge zones [42].

The geological formations in the region range from the Permian to Quaternary, including the Jurassic, Triassic, and Permian strata [35]. Jurassic formations, such as the Penglaizhen (*J*_3_*p*), Suining (*J*_3_*sn*), Shaximiao (*J*_2_*s*), and Ziliujing (*J*_1–2_*z*) formations, consist mainly of sandstone, mudstone, and shale, and are widely distributed in the valley areas. In contrast, the Triassic and Permian formations, represented by the Xujiahe (*T*_3_*xj*), Leikoupo (*T*_2_*l*), Feixianguan (*T*_1_*f*), Jialingjiang (*T*_1_*j*), Longtan (*P*_2_*l*), and Maokou (*P*_1_*m*) formations, are composed mainly of carbonate rocks such as dolomite and limestone, forming the principal lithology of the ridge zones [5]. Structurally, the ridges are characterized by a series of anticlines, which significantly control the regional hydrogeological framework.

The groundwater system in the area exhibits clear spatial heterogeneity. In the ridge regions, groundwater mainly occurs in carbonate aquifers with well-developed fissures and karst conduits, where recharge is primarily derived from vertical infiltration of precipitation and partially from lateral inflow of surface water [5,43]. Groundwater tends to move vertically along fractures and faults, eventually discharging as springs or through subsurface drainage into the Yangtze River. In contrast, the valley zones are underlain by Jurassic clastic rocks that form low-permeability fractured bedrock aquifers with shallow groundwater tables, characterized by local recharge and discharge. The Xujiahe Formation acts as a semi-confining layer between the carbonate and clastic aquifers. The strong geomorphological and lithological contrast between the ridges and valleys exerts a major influence on groundwater flow, storage, and vulnerability to contamination in the urbanized basin.

## 3. Methods and Materials

### 3.1. Field Sampling and Laboratory Analysis

A total of 37 groundwater samples were collected from all monitoring wells within the study area in Chongqing in May 2020, and their spatial distribution is displayed in Figure 1b. To ensure the representativeness and reliability of the collected samples, sampling was primarily conducted during the dry season to avoid groundwater fluctuations and consequent variations in chemical parameters caused by rainfall. Moreover, strict standards were followed at every stage of sample preservation and laboratory analysis. The geographical coordinates of each sampling point were determined using a handheld GPS unit. The samples were mainly collected from phreatic aquifers at depths of 20–30 m. To avoid the influence of stagnant water, groundwater was continuously pumped for more than 30 min before sampling. On-site parameters, including pH and total dissolved solids (TDSs), were measured using a portable WTW Multi 3400i meter (WTW, Weilheim, Germany). Each sampling bottle was rinsed at least three times with the corresponding groundwater before use, and samples were filtered through 0.45 μm membranes. 250 mL of groundwater from each site was collected in polyvinyl chloride (PVC) containers, which were filled to minimize air exposure. The collected samples were then stored at around 3 °C and delivered to a certified laboratory within one week for further analysis.

The major cations (K^+^, Na^+^, Ca^2+^, and Mg^2+^) were quantified using inductively coupled plasma optical emission spectrometry (ICP-OES; Agilent iCAP 7400 DUO, Santa Clara, CA, USA), whereas Cl^−^, SO_4_^2−^, NO_2_^−^, and NO_3_^−^ concentrations were determined by ion chromatography (M-IC, Metrohm, Herisau, Switzerland). HCO_3_^−^ and total hardness (TH) were analyzed by titration. For ICP-OES and ion chromatography, external multi-point calibration curves (typically 5–7 levels) were established using commercially available multi-element standard solutions supplied by Merck KGaA, Darmstadt, Germany (for ICP-OES) and Sigma-Aldrich, St. Louis, MO, USA (for ion chromatography). Instrument calibration was verified every 10 samples by re-measuring a mid-level standard, and recalibration was performed when the deviation exceeded 5%. To maintain analytical precision, quality assurance and control (QA/QC) protocols were rigorously applied, incorporating procedural blanks, certified reference materials (CRMs) and routine replicate analyses. A certified groundwater reference material (GBW(E), NCRM, Beijing, China) was used to check accuracy. Recovery efficiencies for all target ions ranged between 85% and 110%, and the relative standard deviation (RSD) of duplicate measurements (10% of the total samples) was below 5%. Furthermore, the charge balance error (CBE) between anions and cations was consistently kept within ±5%, as calculated by Equation (1).

(1) $$\%\mathrm{CBE}=\frac{\sum \;\mathrm{Cations}\;-\;\sum \;\mathrm{Anions}}{\sum \;\mathrm{Cations}+\;\sum \;\mathrm{Anions}}$$

### 3.2. Inverse Distance Weighting Method

To map the spatial distribution of hydrochemical parameter concentrations, inverse distance weighting (IDW) interpolation was applied. IDW is a deterministic local interpolator that assumes closer samples exert greater influence than more distant ones and is widely used in hydrological and water quality studies, particularly when the sampling network is relatively sparse and non-stationary. To evaluate the performance of the IDW interpolation, leave-one-out cross-validation was conducted by iteratively removing each sampling point and predicting its value based on the remaining data points. The root-mean-square error (RMSE) and mean error between observed and predicted values were calculated to assess whether the IDW interpolation provides an accurate and reliable representation of the spatial patterns of hydrochemical parameters.

(2) $$\mathrm{RMSE}=\sqrt{\frac{\sum_{i=1}^{n}{\left(\bar{z}(s_{i})-z(s_{i})\right)}^{2}}{n}}$$

(3) $$\mathrm{mean}\;\mathrm{error}=\frac{\sum_{i=1}^{n}\left(\bar{z}(s_{i})-z(s_{i})\right)}{n}$$

where $\bar{z}$ donates the mean concentrations of hydrochemical parameters and n donates the number of groundwater samples.

### 3.3. Saturation Index

The mineral saturation index (SI) is used to evaluate the dissolution–precipitation equilibrium between groundwater and aquifer minerals [44]. SI is calculated by comparing the ionic activity product (IAP) of a mineral dissolution reaction with its thermodynamic solubility product constant (Ksp) under the corresponding temperature, pressure, and chemical conditions (Equation (4)). When SI > 0, groundwater is supersaturated and the mineral tends to precipitate; SI = 0 indicates equilibrium; and SI < 0 indicates undersaturation and a tendency for mineral dissolution. Ion activities and equilibrium reactions were computed using the PHREEQC hydrogeochemical model.

(4) $$SI=\log(\frac{\mathrm{IAP}}{K_{\mathrm{sp}}})$$

where SI is the saturation index of the mineral, IAP denotes the ionic activity product, and $K_{\mathrm{sp}}$ represents the thermodynamic solubility product constant.

### 3.4. Human Health Risk Assessment

#### 3.4.1. Deterministic Risk Assessment Model

The non-carcinogenic risks are quantitatively assessed using the human health risk assessment model proposed by the United States Environmental Protection Agency (USEPA) [22]. The evaluation primarily focuses on the non-carcinogenic health risks of groundwater contaminants through oral ingestion in the study. The chronic daily intake (CDI) of the evaluated potentially toxic elements is calculated using Equation (5), and the corresponding parameters are presented in Table 1.

(5) $$\mathrm{CDI}=\frac{C_{w}\;\times \;\mathrm{IR}\;\times \;\mathrm{EF}\;\times \;\mathrm{ED}}{\mathrm{BW}\;\times \;\mathrm{AT}}$$

where CDI represents the average exposure dose through long-term water ingestion (mg/kg·d). Cw denotes the concentration of a given chemical in water (mg/L), while IR indicates the daily groundwater intake rate (L/d). EF corresponds to the exposure frequency (days per year), and ED describes the exposure duration, referring to the total number of years an individual consumes the water (a). BW stands for the average body weight (kg). The average exposure time (AT) is determined as the product of ED and 365 days per year.

The pollutant hazard Index (HI) is calculated by Equation (6).

(6) $$HI=\sum_{i=1}^{n}\frac{{\mathrm{CDI}}_{i}}{RfD}$$

where *RfD* denotes the reference dose for individual pollutants (mg/kg·d).

#### 3.4.2. Probabilistic Risk Assessment Model

To overcome the limitations of the deterministic risk assessment model, in which key exposure parameters such as IR, ED and EF are treated as fixed point values and their variability is ignored, the Monte Carlo Simulation (MCS) is employed in this study. MCS is a numerical technique grounded in probability theory and statistics, whose fundamental principle is to reproduce the behavioral characteristics of complex systems through random sampling from specified probabilistic models. In this model, each uncertain exposure parameter (e.g., IR, ED, EF and BW) is assigned an appropriate probability distribution (such as normal, lognormal or uniform), and Monte Carlo simulations are performed with thousands of independent iterations to propagate parameter variability and generate a probability density function of the resulting risk values. This probabilistic framework provides a more robust quantification of uncertainty than deterministic calculations based on single-point estimates. The suitability of the selected probability distributions is evaluated using the Kolmogorov–Smirnov goodness-of-fit test, and the distributions of the uncertain exposure parameters for different population groups are summarized in Table 2.

The kernel density estimation (KDE) technique is adopted to perform nonparametric estimation, and its corresponding expressions are given as Equations (7) and (8). A Gaussian kernel was adopted (Equation (6)) because of its smoothness and favorable theoretical properties [39]. The bandwidth *h*, which controls the trade-off between bias and variance of the estimator, was initially determined using Silverman’s rule-of-thumb (Equation (9)). To ensure robustness, the sensitivity of the KDE to the bandwidth was examined by varying *h* within a reasonable range (0.8–1.2 h). It is found that the resulting probability curves and high-percentile risk estimates (e.g., the 95th percentile) were only marginally affected. The adequacy of the KDE representation was validated by comparing the estimated densities with the empirical histograms and cumulative distribution functions of the simulated risk values, confirming that the KDE captured the overall shape and tail behavior of the risk distribution without over-smoothing.

(7) $${\hat{f}}_{h}\left(x\right)=\frac{1}{\mathrm{nh}}\sum_{i=1}^{n}\;K\left(\frac{x-x_{i}}{h}\right)$$

(8) $$K(u)=\frac{1}{\sqrt[{}]{2\pi }}exp\left(-\frac{u^{2}}{2}\right)$$

(9) $$h=1.06\sigma n^{-0.2}$$

where ${\hat{f}}_{h}\left(x\right)$ donates the KDE, $K\left(u\right)$ donates the kernel functions, and σ and n denote the sample standard deviation and the number of simulated risk values, respectively.

### 3.5. Research Framework

**Figure 2:**
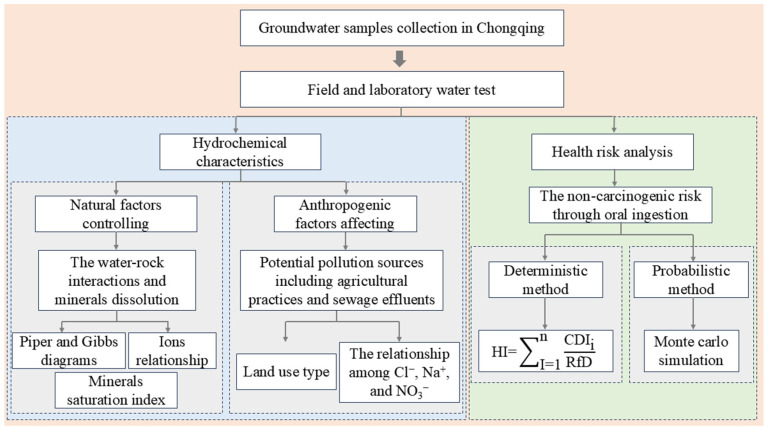
Research framework of the study.

Based on groundwater samples collected in Chongqing, this study identified the driving factors influencing groundwater chemistry and assessed their associated human health risks. Natural factors controlling groundwater chemistry were analyzed using Piper diagrams, Gibbs diagrams, specific ion ratios, and mineral saturation indices. Anthropogenic influences were evaluated through hydrochemical diagrams integrated with land use type analysis. To assess potential human health risks, both deterministic and probabilistic approaches were applied. The overall framework of the study is illustrated in Figure 2.

## 4. Hydrochemical Results

**Figure 3:**
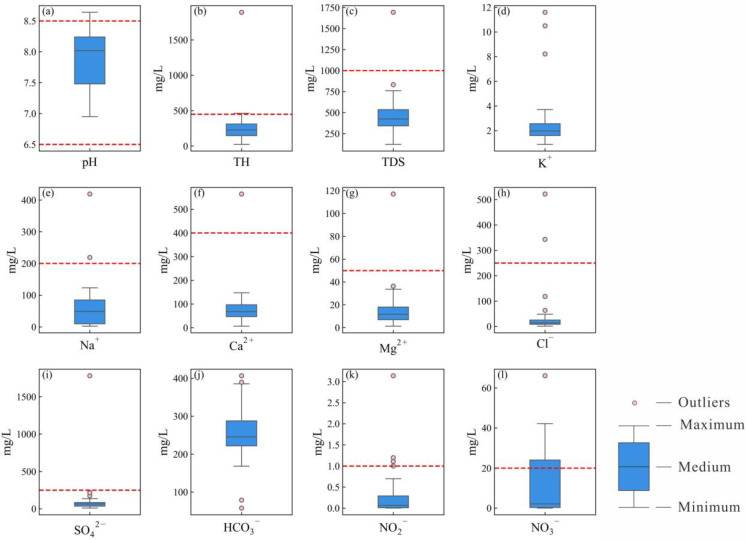
The boxplots of hydrochemcial parameters (the red line donates threshold recommended by China and WHO): (**a**) pH, (**b**) TH, (**c**) TDS, (**d**) K^+^, (**e**) Na^+^, (**f**) Ca^2+^, (**g**) Mg^2+^, (**h**) Cl^−^, (**i**) SO_4_^2−^, (**j**) HCO_3_^−^, (**k**) NO_2_^−^ and (**l**) NO_3_^−^.

**Figure 4:**
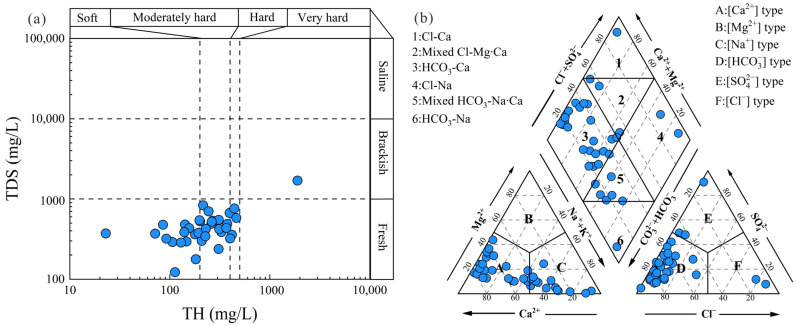
Hydrochemical analysis diagrams: (**a**) Classification plot by TDS vs. TH; (**b**) Piper trilinear diagram.

The statistical results of hydrochemical parameters are presented in Figure 3. The pH values ranged from 7.0 to 8.6, with a mean of 7.9, indicating that groundwater in the main urban area of Chongqing is predominantly neutral to slightly alkaline. Total hardness (TH) and total dissolved solids (TDSs) varied from 22.88 mg/L to 1891.24 mg/L and from 122 mg/L to 1694 mg/L, with average concentrations of 280.79 mg/L and 470.53 mg/L, respectively. Only one groundwater sample exceeded the drinking water quality threshold for TH and TDS as recommended by the Chinese Standard for Groundwater Quality (GB/T 14848–2017, Class III) [45]. The classification concerning TH varies from soft water to very hard, while it varies from fresh water to brackish water (Figure 3a). The average concentrations of cations ranked as Ca^2+^ (85.58 mg/L) > Na^+^ (61.47 mg/L) > Mg^2+^ (16.28 mg/L) > K^+^ (2.68 mg/L). The concentration ranges for these cations were from 6.47 mg/L to 564.41 mg/L, 2.26 mg/L to 419 mg/L, 1.25 mg/L to 117.10 mg/L and 0.88 mg/L to 11.6 mg/L for Ca^2+^, Na^+^, Mg^2+^ and K^+^, respectively. For anions, the mean concentrations decreased in the sequence: HCO_3_^−^ (251.85 mg/L) > SO_4_^2−^ (115.51 mg/L) > Cl^−^ (41.49 mg/L) > NO_3_^−^ (11.88 mg/L) > NO_2_^−^ (0.29 mg/L). Thus, the dominant hydrochemical types of groundwater were categorized as HCO_3_-Ca (Figure 4). Recommended by the Chinese Standard for Groundwater Quality [1], the concentrations of NO_3_^−^ exhibited the highest proportions (27.03 mg/L), exceeding the threshold (1 mg/L), followed by the concentrations of NO_2_^−^, with an exceeded proportion of 8.11%.

To further investigate the similarities and differences in the spatial distribution patterns of various hydrochemical parameters, the inverse distance weighting (IDW) method was employed with ArcGIS Pro 3.0. The interpolation results for each hydrochemical parameter were validated using the cross-validation method. The validation outcomes are summarized in Table 3. The RMSE values for all hydrochemical parameters are very small, and the corresponding mean errors are close to zero. This indicates that the IDW predictions do not exhibit systematic overestimation or underestimation and that the interpolation errors are negligible compared with the observed concentration ranges of each parameter.

**Figure 5:**
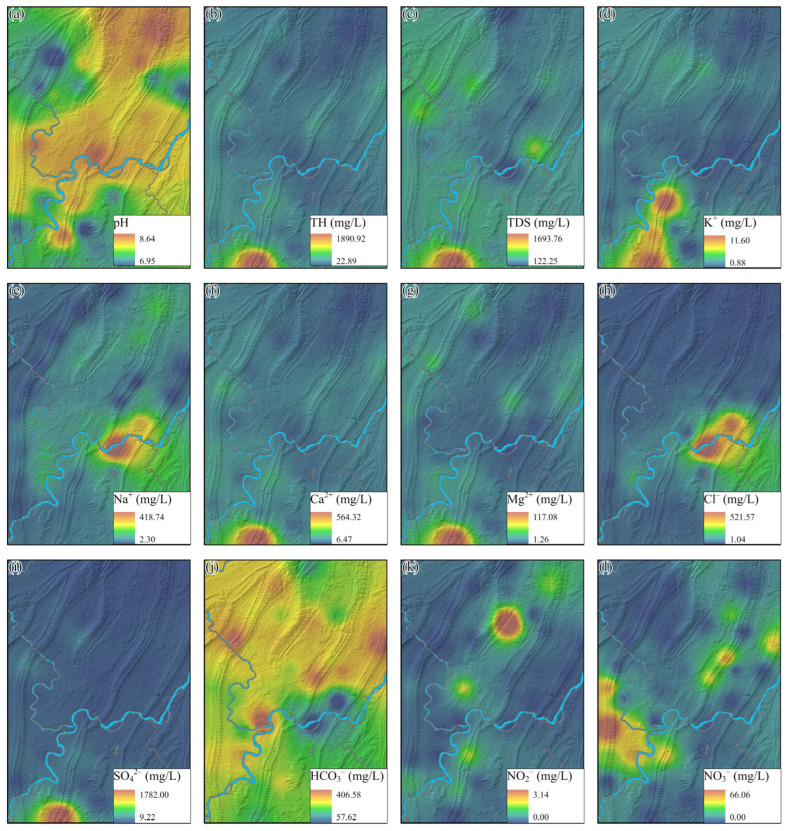
The spatial distribution of each hydrochemical parameter in the study area: (**a**) pH, (**b**) TH, (**c**) TDS, (**d**) K^+^, (**e**) Na^+^, (**f**) Ca^2+^, (**g**) Mg^2+^, (**h**) Cl^−^, (**i**) SO_4_^2−^, (**j**) HCO_3_^−^, (**k**) NO_2_^−^ and (**l**) NO_3_^−^.

The spatial distributions of each hydrochemical parameter were illustrated in Figure 5. Groundwater samples with a slightly alkaline characteristic are mainly situated in the north and central areas. Notably, the hydrochemical parameters of TH, TDS, K^+^, Ca^2+^, Mg^2+^, and SO_4_^2−^ exhibited a similar spatial distribution, with outlier values scattered in the southern part of the study area (Figure 5b–d,f,g,i). The unique groundwater location with elevated concentrations was identified near a geothermal water discharge site, where extensive dissolution of evaporites has occurred [2]. High concentrations of Ca^2+^ and SO_4_^2−^ in groundwater originate from the geothermal water, contributing to increased levels of TH and TDS. Moreover, one outlier site of groundwater sample with elevated Na^+^ and Cl^−^ concentrations is distributed in the central area, possibly resulting from the water–rock interactions in the sandstone-mudstone sequences. Elevated concentrations of nitrite and nitrate in groundwater are predominantly observed in the northern and western portions of the study area, with a maximum concentration of 3.14 mg/L and 66.06 mg/L, respectively, both exceeding three times the regulatory standards.

## 5. Discussion

### 5.1. Natural Factors Controlling Groundwater Chemistry

**Figure 6:**
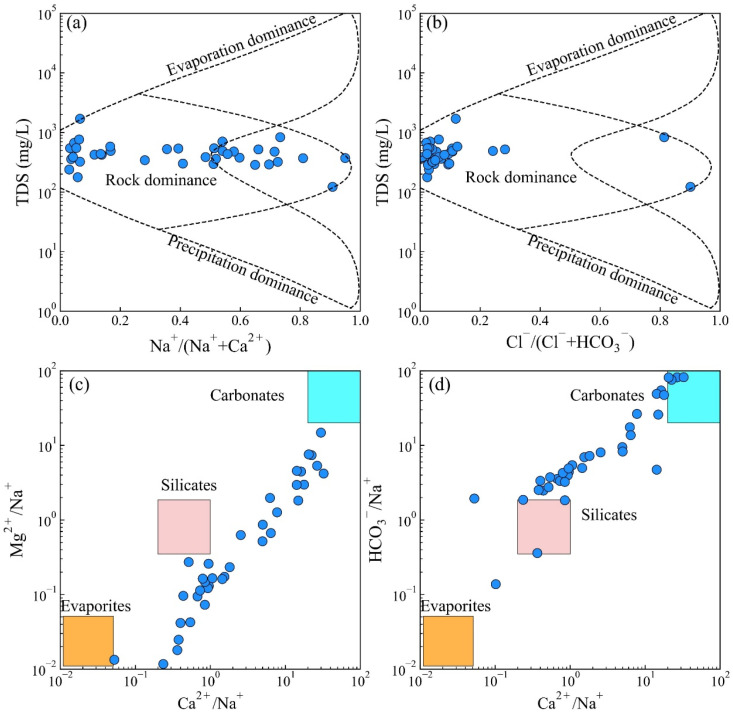
The Gibbs diagrams (**a**) TDS vs. Na^+^/(Na^+^ + Ca^2+^) and (**b**) TDS vs. Cl^−^/(Cl^−^ + HCO_3_^−^). Gaillardet diagrams: (**c**) Mg^2+^/Na^+^ vs. Ca^2+^/Na^+^ and (**d**) HCO_3_^−^/Na^+^ vs. Ca^2+^/Na^+^.

A Gibbs diagram is a widely used tool for identifying the source processes of evaporation, water–rock interaction, and precipitation [3]. In the main city of Chongqing, water–rock interaction controls the primary natural process, with plenty of samples scattered in the rock dominance zone (Figure 6a,b). The Gaillardet diagrams are further applied in the discovery of the dominant rock types of water–rock interaction. The specific diagrams divided the dominant rock types into three categories (evaporites, silicates, and carbonates) concerning the ratios of Ca^2+^/Na^+^, Mg^2+^/Na^+^, and HCO_3_^−^/Na^+^. A small proportion of groundwater samples were plunged near the evaporites endmember, while most of the groundwater samples were situated between the silicate endmember and the carbonates endmember. The results revealed a mixed control by silicate weathering and carbonate dissolution.

**Figure 7:**
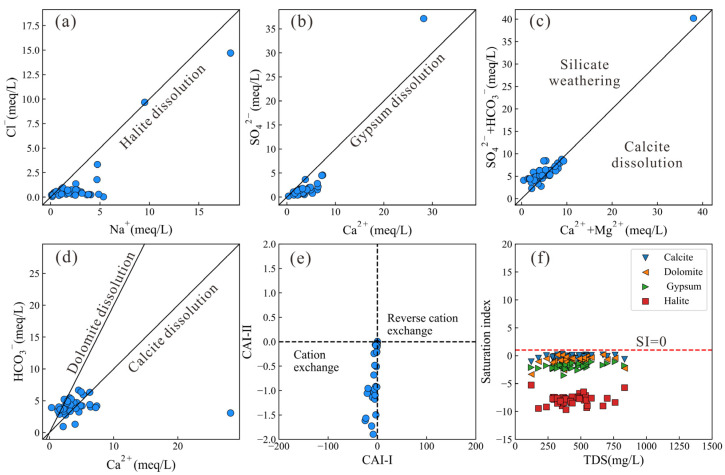
The scatter plots of (**a**) Cl^−^ vs. Na^+^, (**b**) SO_4_^2−^ vs. Ca^2+^, (**c**) HCO_3_^−^ + SO_4_^2−^ vs. Ca^2+^ + Mg^2+^, (**d**) HCO_3_^−^ vs. Ca^2+^, (**e**) CAI II vs. CAI I, and (**f**) Saturation index vs. TDS.

Moreover, the specific ion ratios of major ions could further help to identify the roles of the minerals in controlling groundwater chemistry [46,47]. When the ratios of Cl^−^/Na^+^ and SO_4_^2−^/Ca^2+^ are close to 1:1, the dissolution of halite and gypsum predominantly controls the concentrations of Cl^−^, Na^+^, and SO_4_^2−^, Ca^2+^ in groundwater, respectively (Equations (10) and (11)). As shown in Figure 7a,b, some groundwater samples plot along the 1:1 line, while others fall below it. This indicates that, in addition to halite and gypsum dissolution, other hydrochemical processes also contribute to the enrichment of Na^+^ and Ca^2+^ in groundwater. Notably, a sampling site with elevated concentrations of Cl^−^ and Na^+^ was observed near the center of the study area, located within sandstone-mudstone sequences. Intense silicate weathering may account for this phenomenon. In contrast, the sampling site with elevated concentrations of SO_4_^2−^ and Ca^2+^ scattered above the 1:1 line, suggesting limited influence from gypsum dissolution. As previously mentioned, the high concentrations of SO_4_^2−^ and Ca^2+^ may be derived from the geothermal water recharge. The ratio of HCO_3_^−^ + SO_4_^2−^ vs. Ca^2+^ + Mg^2+^ can be used to distinguish between water–rock interactions involving silicate weathering and calcite dissolution. As shown in Figure 7c, both silicate weathering and calcite (a carbonate mineral) dissolution contribute to groundwater chemistry, which is consistent with the results revealed by the Gaillardet diagrams. Furthermore, the dissolution of dolomite and calcite can lead to distinct HCO_3_^−^/Ca^2^ ratios in groundwater. An HCO_3_^−^/Ca^2+^ ratio of 1 is expected when calcite dissolution dominates, whereas a ratio of approximately 0.5 suggests dolomite dissolution as the primary process. Figure 7d showed that both dolomite and calcite dissolution contributed to the enrichment of HCO_3_^−^ and Ca^2+^ in groundwater.

(10) $$\mathrm{NaCl}\;\to \;Na^{+}+Cl^{-}$$

(11) $$\mathrm{CaS}O_{4}\cdot 2H_{2}O\;⇌\;Ca^{2+}+SO_{4}^{2-}+2H_{2}O$$

(12) $$\mathrm{CAI}–I=\left(Cl^{-}\;-\;\left({\mathrm{Na}}^{+}+K^{+}\right)\right)/Cl^{-}$$

(13) $$\mathrm{CAI}–\mathrm{II}=\left(Cl^{-}\;-\;\left({\mathrm{Na}}^{+}+K^{+}\right)\right)/\left(\mathrm{HC}O_{3}^{-}+SO_{4}^{2-}+CO_{3}^{2-}+NO_{3}^{-}\right)$$

(14) $${\mathrm{Ca}}^{2+}+2\mathrm{NaX}\;⇌\;2{\mathrm{Na}}^{+}+{\mathrm{CaX}}_{2}$$

The cation exchange process could be normally identified by the chlor-alkali indices (CAIs) and can be categorized as a cation exchange process and a reverse cation exchange process (Equations (12)–(14)). The Ca^2+^ in groundwater would be replaced by the Na^+^ in the surrounding rock or clay minerals when the cation exchange process occurs. In contrast, Na^+^ in groundwater would be replaced by the Ca^2+^ in the surrounding rock or clay minerals when the reverse cation exchange process occurs. In the main urban area of Chongqing, the cation exchange process dominated, leading to an increasing concentration of Na^+^. Saturation index (SI) of minerals could further distinguish the effects of normal minerals in the water interaction process. Figure 7f illustrates that SI values of all minerals (calcite, dolomite, gypsum, and halite) were below zero, showing that all minerals participated in the hydrochemical process.

Generally, most samples fall within the silicate–carbonate mixing domain, indicating that carbonate (dolomite and calcite) dissolution and silicate weathering are the dominant processes at the regional scale, while evaporite dissolution plays a secondary or localized role. Although the bedrock is primarily composed of limestones and red-bed sandstones, evaporitic components—such as gypsum, halite cements, and thin interbeds—within the red beds serve as a plausible source of dissolved salts. From a hydrochemical perspective, near-unity Na^+^/Cl^−^ equivalent ratios, coupled increases in Ca^2+^ and SO_4_^2−^ concentrations, and negative saturation indices for halite and gypsum collectively indicate active dissolution of evaporite minerals.

### 5.2. Anthropogenic Factors Affecting Groundwater Chemistry

**Figure 8:**
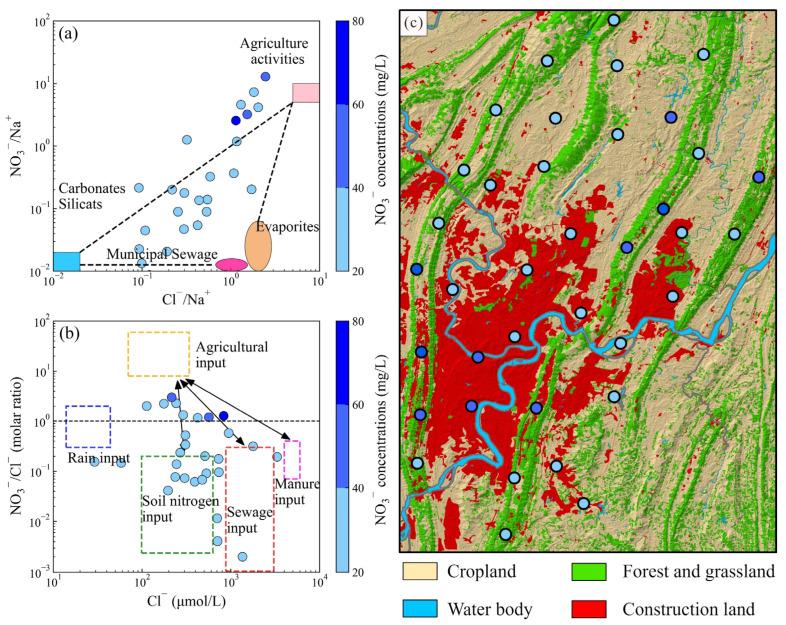
(**a**) NO_3_^−^/Na^+^ vs. Cl^−^/Na^+^, (**b**) NO_3_^−^/Cl^−^ (molar ratio) vs. Cl^−^ (μmol/L), and (**c**) the land use type of the study area.

In the main urban areas of Chongqing, construction land dominates the land use types, while cropland is present in adjacent areas (Figure 8c). Thus, the impact resulting from the urban and agricultural activities can not be neglected. Concerning the exceeded proportions of each hydrochemical parameter, the point source pollution of nitrite and nitrate was found in the northern and western portions of the study area. The NO_2_^−^ pollution could correspond to the industrial activities where there is an industry situated at the sampling site. Moreover, a thorough analysis of nitrate pollution should be conducted to address its point pollution. Chloride is a conservative ion that is not affected by chemical, physical, or biological changes in the hydrological system and is commonly utilized for nitrate pollution identification [48]. As shown in Figure 8a,b, the dispersive groundwater samples were situated in various endmembers in the main urban areas of Chongqing. Groundwater samples with elevated nitrate concentrations (more than 40 mg/L) were scattered in cropland and construction land, further demonstrating the impacts of agricultural activities, sewage, and manure input on groundwater chemistry. Moreover, the soil nitrogen was also identified as a nitrate source (Figure 8). Notably, the highest nitrate concentration was situated in the cropland area, further signifying that agricultural activities are the key factors contributing to nitrate pollution in groundwater. Moreover, previous studies have also demonstrated that chemical fertilizer is the primary source of nitrate nitrogen in the Chongqing region, followed by domestic sewage and manure, soil nitrogen, and atmospheric deposition [42]. Intensive agricultural activities have contributed to elevated background concentrations of nitrate nitrogen in local groundwater within the urban area of Chongqing.

### 5.3. Evaluation of Human Health Risk

#### 5.3.1. Deterministic Evaluation of Human Health Risk

The nitrite and nitrate in groundwater were recognized as the noncarcinogenic elements recommended by USEPA [22]. Thus, the human health risks caused by the nitrite and nitrate in the main urban area of Chongqing were determined by the HHR model. Hazard quotient (HQ) caused by NO_3_^−^ for children and adults ranged from 0 to 2.93 and 0 to 1.00, with a mean value of 0.53 and 0.18, respectively. 27.02% of groundwater samples were found to have a risk for children in the west of the study area. A few groundwater samples (2.70%) were determined with the noncarcinogenic risk for adults. Similarly, the areas with high risk are also situated in the west of the study area. The HQ caused by NO_2_^−^ ranged from 0.00 to 2.22 and 0.00 to 0.76 for children and adults, with an average value of 0.20 and 0.07, respectively. The high risks caused by NO_2_^−^ exhibited a point pollution in the area where the elevated NO_2_^−^ concentrations were identified in the northern area of the main urban city. The hazard index (HI) combined the hazard quotient (HQ) caused by nitrate and nitrite, ranging from 0.00 to 3.02 and from 0.00 to 1.04 for children and adults, with a mean value of 0.73 and 0.25, respectively. 29.02% and 2.70% of groundwater samples were found with a risk for children and adults, respectively. Spatially, the high-risk areas of the HI for children and adults are the ensemble of each hazard quotient, mainly distributed in the north and the west of the study area.

**Figure 9:**
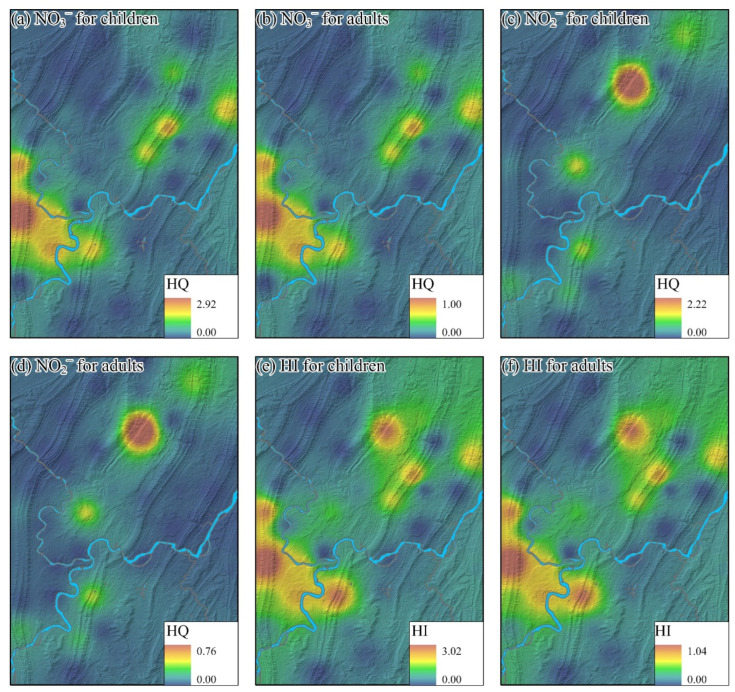
The spatial distribution of the human health risk: the hazard quotient (HQ) of NO_3_^−^ for children and adults (**a**,**b**), the hazard quotient (HQ) of NO_2_^−^ for children and adults (**c**,**d**) and the hazard index (HI) for children and adults (**e**,**f**).

Overall, the deterministic evaluation of human health risk indicated that children may suffer from the noncarcinogenic risk caused by nitrate and nitrite, particularly in the northern and western areas of the main urban city (Figure 9).

#### 5.3.2. Probabilistic Assessment of Human Health Risk

**Figure 10:**
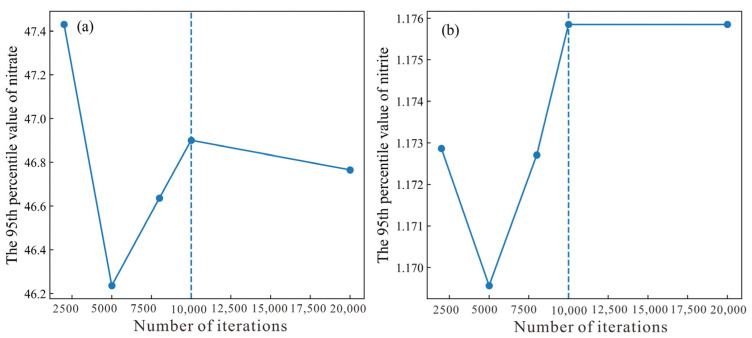
The 95th percentile value of (**a**) nitrate and (**b**) nitrite.

Previous studies have demonstrated the robustness of the kernel density method (KDE) to enhance the Monte Carlo simulation compared with the parametric method, particularly in the simulations of groundwater hydrochemical parameters. Thus, in our study, the KDE method was applied in the simulations of NO_3_^−^ and NO_2_^−^ concentrations. To assess the numerical stability of the KDE-based Monte Carlo simulations, simulations were performed with 2000, 5000, 8000, 10,000, and 20,000 iterations, and the corresponding 95th percentile estimates were compared (Figure 10). The 95th percentile values exhibited only negligible variation when the number of iterations increased from 10,000 to 20,000. Furthermore, the mean relative changes in the estimated mean concentration and exceedance probability between 10,000 and 20,000 iterations were 0.085% and 1.922% for the concentrations of nitrate and nitrite, respectively, both within acceptable tolerance levels. These results indicate that numerical convergence was achieved and support the use of 10,000 iterations as a stable and sufficient sample size for the final analysis.

**Figure 11:**
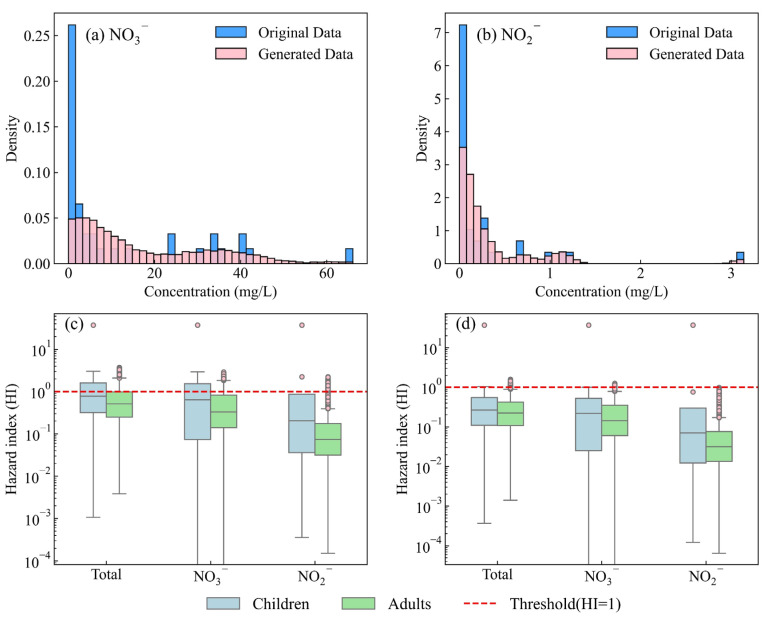
The diagrams of original data sets and generated data sets (**a**) NO_3_^−^ and (**b**) NO_2_^−^, and the results of HI calculated by original data sets (**c**) and generated data sets (**d**).

The generated datasets of the two parameters are illustrated in Figure 11. The generated NO_3_^−^ and NO_2_^−^ concentrations ranged from 0.00 mg/L to 66.08 mg/L and 0.00 mg/L to 3.14 mg/L, with a mean value of 17.53 mg/L and 0.35 mg/L, respectively. The generated data demonstrates a close alignment with the distribution of the original dataset. Moreover, the significance tests of both datasets were less than 0.05 (0.0000 and 0.0006 for nitrate and nitrite, respectively) and effectively address the uncertainties associated with limited sampling.

A simulation of exposure parameters was performed based on predefined distribution types and their corresponding parameter values, resulting in the creation of 10,000 stochastic datasets [6]. Thus, the hazard quotient corresponding to NO_3_^−^ and NO_2_^−^ was calculated. The hazard quotient (HQ) of NO_3_^−^ varied from 0.00 to 2.90 and 0.00 to 1.26 for children and adults, with a mean value of 0.51 and 0.22, respectively. 0.18% of the samples exceeded the threshold for children, while no samples exceeded the threshold for adults. The hazard quotient (HQ) of NO_2_^−^ varied from 0.00 to 2.23 and 0.00 to 0.99 for children and adults, with a mean value of 0.16 and 0.07, respectively. 0.02% of the samples exceeded the threshold for children, while no samples exceeded the threshold for adults. As for the HI, the values ranged from 0.00 to 3.73 and from 0.00 to 1.59, with an average value of 0.67 and 0.29 for children and adults, respectively. 0.26% of the samples exceeded the threshold for children, while 0.01% samples exceeded the threshold for adults. The simulation results derived from the KDE-based uncertainty model exhibit a close correspondence with those obtained from the deterministic model (Figure 11). The minor discrepancies observed can be primarily attributed to the limited sample size. In estimating the probability density curve, the KDE method employs a smoothing kernel that extends the influence of individual data points across neighboring regions, thereby enhancing their contribution to the overall distribution [39].

**Figure 12:**
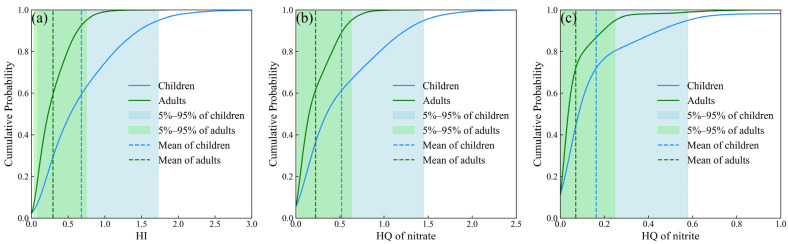
Cumulative probability distribution curves for (**a**) HI, (**b**) for nitrate and (**c**) for nitrite.

To evaluate the reliability of the proposed approach, a Monte Carlo simulation incorporating the kernel density estimation (KDE) method was conducted to compute the 95% confidence intervals (CIs) for each group and each non-carcinogenic element (Figure 12). As presented in Table 4, all group means and element-specific means fall within the corresponding 95% CIs derived from the KDE-based Monte Carlo simulation, confirming the high reliability of the method. Additionally, a bootstrap resampling approach was employed to rigorously assess the uncertainty associated with the mean differences, thereby strengthening the robustness of the statistical inference. The 95% CIs for children were −0.030 to 0.488 (NO_3_^−^) and −0.005 to 0.124 (NO_2_^−^), while those for adults were −0.140 to 0.618 (NO_3_^−^) and −0.003 to 0.197 (NO_2_^−^), all of which include zero, indicating no statistically significant difference between the groups.

### 5.4. Perspectives and Limitations

In this study, natural and anthropogenic factors influencing groundwater chemistry were identified, and the human health risks associated with nitrate and nitrite were assessed in the urban area of Chongqing. Globally, numerous studies have also focused on human health risks related to potentially harmful elements in groundwater. For instance, He found that a combination of geogenic and industrial sources of heavy metals dominated health risks in Zhengzhou, China [49]. Labad identified sewage system leaks and surface water infiltration as the primary contributors to groundwater contamination in Barcelona, Spain [41]. Sharma reported that children are particularly vulnerable to potential health threats in Delhi, India [40]. These findings collectively indicate that combined geogenic and anthropogenic sources contribute to groundwater-related health risks, and that children are especially susceptible to such risks in urban areas worldwide.

However, several limitations should be acknowledged:

(1) Only 37 groundwater samples were collected in May 2020. The spatial heterogeneity of groundwater chemical parameters may therefore not have been fully captured, especially in areas with intensive human activities and complex geological conditions. Moreover, the lack of groundwater samples from the wet season prevents the temporal variability of groundwater and its driving factors over an annual cycle from being adequately revealed. Future research should expand the sampling scale and enhance the spatiotemporal resolution of monitoring to enable a more comprehensive assessment and management of groundwater quality and health risks.
(2) In this study, only the human health risks associated with nitrate and nitrite were quantified. Potential risks related to heavy metals were not considered because of the analytical methods selected for water-quality testing. Future work will involve comprehensive analyses of heavy metals at the same sampling sites to evaluate their health risks and identify their possible sources, thereby contributing to a more complete understanding of chemistry-related health risks in Chongqing’s urban groundwater system. Moreover, the sources of nitrite were not identified in this study. Further research could incorporate positive matrix factorization (PMF) to elucidate the sources of nitrite in groundwater.
(3) The spatial patterns of groundwater quality and health risk were interpolated using a deterministic IDW method. Although cross-validation indicated acceptable predictive performance, IDW does not explicitly account for spatial autocorrelation structures or provide interpolation uncertainty, and it cannot represent groundwater flow and solute-transport processes. In addition, no groundwater flow or transport modeling was conducted in this study, so the migration pathways, travel times and future evolution of nitrate contamination remain insufficiently constrained. Future studies should compare alternative interpolation techniques (e.g., kriging or co-kriging) and couple them with physically based flow and transport models to better characterize the spatial–temporal dynamics and sources of groundwater pollution.

## 6. Conclusions

In this study, a total of 37 groundwater samples were collected in the main urban areas of Chongqing to investigate the driving factors controlling groundwater chemistry and the human health risks associated with groundwater use. The main findings are as follows:

(1) Groundwater in the main urban areas of Chongqing exhibited neutral to slightly alkaline conditions. The cations ranked as Ca^2+^ > Na^+^ > Mg^2+^ > K^+^ while the anions decreased in the sequence: HCO_3_^−^ > SO_4_^2−^ > Cl^−^ > NO_3_^−^ > NO_2_^−^. HCO_3_^−^ Ca was identified as the dominant hydrochemical facies.
(2) Silicate weathering and carbonate (dolomite and calcite) dissolution dominated the natural process. The evaporite dissolution played a secondary or localized role in controlling groundwater chemistry. Industrial and agricultural activities corresponded to the elevated concentrations of NO_3_^−^ and NO_2_^−^ in groundwater.
(3) 29.02% and 2.70% of the groundwater samples posed non-carcinogenic health risks to children and adults, respectively, with HI values exceeding the safety threshold (HI > 1). The generated data from the KDE-based Monte Carlo simulation showed close alignment with the distribution of the original dataset, and all mean values of the original dataset fell within their corresponding 95% confidence intervals (CIs) of the generated data, confirming the high reliability of the method. Furthermore, bootstrap resampling results also indicated no statistically significant difference between the two groups.

Several measures are recommended to enhance groundwater management in Chongqing’s main urban area: establish and maintain a long-term groundwater monitoring network with increased sampling frequency in areas characterized by intensive land use and in proximity to vulnerable populations, such as children; strengthen agricultural management through the promotion of rational fertilizer application, optimization of irrigation practices, and implementation of buffer zones to mitigate nitrate leaching; improve the collection and treatment of domestic sewage and urban runoff, particularly in densely populated districts, to prevent leakage and infiltration into shallow aquifers; and reinforce supervision of geothermal and industrial wastewater discharges by ensuring appropriate treatment and, where applicable, controlled reinjection to prevent localized deterioration of groundwater quality.

## Figures and Tables

**Table 1:** Values of each parameter for human health risk assessment.

Composition	RfDoral (mg/(kg×day))	Exposure Parameter	IR	EF	ED	BW
NO_3_^−^	1.6	Adult	0.85	365	6	12
NO_2_^−^	0.1	Children	1.5	365	30	61.74

**Table 2:** The probability distribution of each exposure parameter.

Parameters	Distribution	
	Children	Adults
IR	N (1.5, 0.15)	N (0.85, 0.09)
BW	LN (61.75, 6.18)	LN (15.0, 1.50)
EF	T (180, 365, 345)	T (180, 365, 345)

Where N donates the normal distributon, LN donates the lognorm distribution and T donates the triangular distribution.

**Table 3:** The performance metrics were obtained using the cross-validation method for IDW interpolation.

Parameter	pH	TDS	TH	K^+^	Na^+^	Ca^2+^	Mg^2+^	Cl^−^	SO_4_^2−^	HCO_3_^−^	NO_2_^−^	NO_3_^−^
RMSE	0.000	0.005	0.001	0.000	0.000	0.006	0.000	−0.007	0.005	−0.005	0.000	0.000
Mean error	0.005	2.890	3.240	0.027	0.857	0.973	0.222	1.062	3.211	0.843	0.007	0.122

**Table 4:** The statistical results of the Monte Carlo simulation and the Bootstrap.

	Children ^a^	Adults ^a^	Children ^b^	Adults ^b^
Mean values of the deterministic model	0.53	0.18	0.20	0.07
95% CI of the Monte Carlo simulation with the KDE method	0.013–1.628	0.006–0.690	0.003–0.690	0.001–0.298
Mean values of Bootstrap	0.219	0.031	0.365	0.111
95% CI of Bootstrap	−0.030–0.488	−0.005–0.124	−0.140–0.618	−0.003–0.197

Note: ^a^ corresponds to the Hazard quotient caused by NO_3_^−^; ^b^ corresponds to the Hazard quotient caused by NO_2_^−^.

## Data Availability

The data presented in this study are available upon request from the corresponding author.
